# Regional variability in craniofacial stiffness: a study in normal and Crouzon mice during postnatal development

**DOI:** 10.1007/s10237-025-01962-7

**Published:** 2025-05-25

**Authors:** Marius Didziokas, Miranda Steacy, Tengyang Qiu, Arsalan Marghoub, Ali Alazmani, Erwin Pauws, Mehran Moazen

**Affiliations:** 1 Department of Mechanical Engineering, University College London, London, UK; 2 School of Mechanical Engineering, University of Leeds, Leeds, UK; 3 Developmental Biology and Cancer Research and Teaching Department, UCL Great Ormond Street Institute of Child Health, University College London, London, UK

**Keywords:** Craniofacial system, Biomechanics, Suture, Craniosynostosis, Mechanobiology

## Abstract

Craniosynostosis (CS) is the premature closure of craniofacial joints known as sutures. Typically, this condition is treated by numerous invasive surgical interventions. Previously we investigated the level of mechanical strain induced due to frontal bone loading on a mouse model of this condition in light of a minimally invasive cyclic bone loading, showing success in retaining coronal suture patency in the Crouzon mouse model. Here we expanded on the previous investigations and characterised the response to external loading on the anterior part of the parietal bone, posterior part of the parietal bone and interparietal bone in addition to the previously investigated frontal bone loading. The results highlighted the significantly higher deformation of the skull and cranial joints during loading of the posterior skull compared to anterior skull loading. These results suggest that loading-based treatment requires different loading regimes depending on location. Additionally, the response of the coronal suture was investigated directly at postnatal day 7 (P7) in both mutant and wild-type animals. The wild-type mice exhibited significant deformation of the coronal suture across all loading locations, whereas no significant deformation was observed in the mutants. Finally, the experimental results were utilised to develop and analyse computational models of WT mice at three ages: P7, P14, and P21. This underscored the challenges in accurately capturing the highly variable response of the mouse craniofacial system to external loading. In summary, this work provided more details on the mechanics of the mouse craniofacial system and its variable overall stiffness across the different anatomical regions of the skull.

**Supplementary Information:**

The online version contains supplementary material available at 10.1007/s10237-025-01962-7.

## Introduction

The vertebrate skull roof is composed of separate bones connected via fibrous soft tissue joints known as cranial sutures. These sutures play a major role in facilitating birth and permit postnatal brain growth (Herring 1972; Opperman 2000; Richtsmeier and Flaherty 2013; Liang et al. 2023, 2024). The sutures either differentiate fully to bone (fuse) or remain unfused leaving micrometre gaps as the brain reaches its maximum size. The fate of the suture depends on a number of factors including the underlying embryonic cellular origin, the species, the individual and the specific suture in question. The fundamental understanding of the mechanobiology of the craniofacial system is limited (Wang and Mao 2002; Mao 2002, 2003; Kopher and Mao 2003; Peptan et al. 2008; Takeshita et al. 2017). In particular, the level of deformation permitted and experienced by the sutures during ab/normal development and its biological impact is still unclear (Tanaka et al. 2000; Kopher et al. 2003; Oppenheimer et al. 2012; Herring et al. 2024). Understanding the role of the sutures in permitting calvarial deformation and development can inform the treatment of craniofacial conditions including craniofacial injuries in adults and congenital conditions in infants.

Premature fusion of the sutures is known as craniosynostosis (CS). The condition affects 1 in 2,000 births, with the incidence increasing 2–3 fold in recent years (van der Meulen et al. 2009; Johnson and Wilkie 2011; Cornelissen et al. 2016; Tønne et al. 2020). Various techniques have been devised by craniofacial surgeons to improve the treatment of this condition (David and Sheen 1990; Jimenez and Barone 1998; Rahimov et al. 2016; Delye et al. 2018; Breakey et al. 2021). In parallel, geneticists and developmental biologists have focused on understanding the genetic components involved in the development of CS. Their investigations have led to the development of a number of animal models presenting the condition (Rice et al. 2003; Eswarakumar et al. 2004; Ishii et al. 2015; Flaherty et al. 2016; Katsianou et al. 2016; Merkuri and Fish 2019; Lee et al. 2019). The Crouzon mouse model (*Fgfr2*^*C342Y/*+^) was developed in 2004, after the discovery of the genes responsible for the Crouzon syndrome in human patients. Crouzon syndrome presents primarily with a premature fusion of the coronal sutures (between the frontal and parietal bones) among other cranial joint abnormalities in both human and mice (Oldridge et al. 1995). The early fusion of the coronal sutures has been associated with a short and domed skull shape in the mouse model (Perlyn et al. 2006; Martínez-Abadías et al. 2013; Liu et al. 2013). The early fusion typically begins at embryonic stages (E18.5) and full closure is commonly achieved by postnatal day 20 (P20). Coronal sutures remain patent in wild-type (WT) mice (Eswarakumar et al. 2004; Peskett et al. 2017).

A previous in vivo study by Moazen et al. (2022) suggested that external dorsoventral cyclic loading of the frontal bone may delay or prevent coronal suture fusion in Crouzon mice. The underlying biological mechanisms of the aforementioned therapeutic effect in the Crouzon mice, i.e. retention of coronal suture patency up to P21 as well as the mechanical effects of the loading remain unclear. Didziokas et al. (2024a) investigated the mechanical effect of the aforementioned loading in terms of deformation and strain induced across the calvarial sutures in a multiscale ex vivo and in vivo study. The latter study developed a novel methodology to non-invasively estimate the suture strain from computed tomography (CT) data. The strains during loading conditions comparable to the in vivo study were estimated at P7, P14 and P21. This has set a strong foundation for future testing of this approach in animal models displaying early fusion of coronal suture. Nonetheless, several questions remain: what level of strain the current loading regime will induce across the sutures and how should the loading regime be applied to other regions of the skull, i.e. targeting other calvarial sutures. Secondly, would computational models based on finite element method be able to accurately predict the level of strain due to such loading in this highly visco-elastic system. These two questions were the main focus of the present study expanding upon the previous study of Didziokas et al. (2024a).

The overall aim of this study was to investigate the response of the mouse craniofacial system to external loading with varied anatomical loading locations experimentally and to compare the experimental results to fully disarticulated computational models. The specific objectives were to investigate the effects of varied loading locations on: (1) the overall skull deformation; (2) the coronal suture gap distance in both Crouzon and WT mice at P7; (3) the calvarial sutures strain for WT mice at P7, P14 and P21 and (4) the predictive capabilities of the developed computational models against the suture strain results.

## Materials and methods

**Fig. 1:**
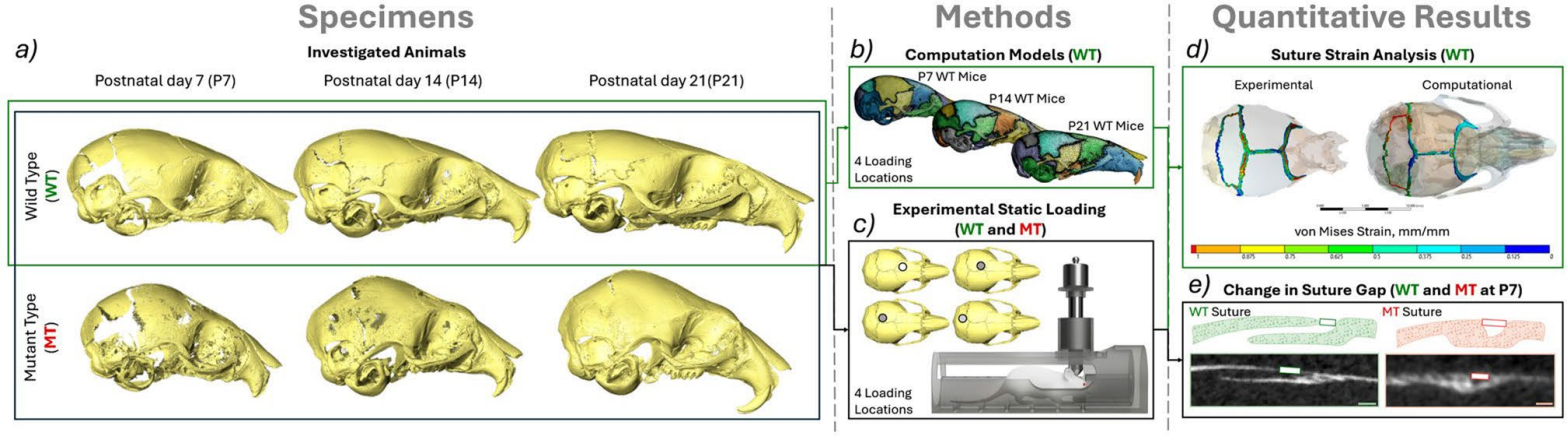
An overview of the overall workflow of this study. **a** Investigated animals—P7(WT *n* = 4, MT *n* = 4), P14(WT *n* = 4, MT *n* = 4) and P21(WT *n* = 4, MT *n* = 4), **b** computational models of WT animals developed from the unloaded scans of the frontal loading at P7, P14 and P21 investigated at 4 different loading locations (frontal bone, anterior part of the parietal bone, posterior part of the parietal bone and interparietal bone), **c** Experimental static loading of both MT and WT animals at P7, P14 and P21 investigated at 4 different loading locations, **d** suture strain analysis of the experimentally loaded animals and comparison to the computational model results and **e** linear measurements of the coronal suture gap in both MT and WT animals at P7 comparing the loaded and unloaded states across 200 slices of the straight region of the suture

The experimental data in objectives (1,2,3) were obtained through a series of ex vivo in situ experiments comparable to the ones carried out in the previous study (Didziokas et al. 2024a) on wild type and Crouzon *Fgfr2*^*C342Y/*+^ mice at P7, P14 and P21. In addition to the loading of the frontal bone, three additional loading locations were considered: the anterior part of the parietal bone, the posterior part of the parietal bone and the interparietal bone. The loadings were carried out a tip diameter (1 mm) away from the midline on the left side of the skull. The computational models for objective (4) were developed based on the CT data of the unloaded frontal bone loading WT mice at each of the investigated ages. A graphical outline of this work is presented in Fig. 1.

### Specimens

Mutant- and wild-type mice were investigated; for the former, the *Fgfr2*^*C342Y/*+^ Crouzon mouse model was used. These were derived by the European Mouse Mutant Archive (EMMA) at MRC Harwell, as described by Peskett et al. (2017). Ex vivo studies were carried out on fresh, refrigerated (4 °C) cadavers, i.e. the analyses were carried out within four days of the animals being culled.

In total 24 cadavers were investigated in this work. (4 P7 WT, 4 P7 MT, 4 P14 WT, 4 P14 MT, 4 P21 WT and 4 P21 MT) With one animal per different type, different age and different loading location. The low number of animals was justified by large differences observed in the mechanical response to different loading locations. Additionally, the variation in the strain results was already established in Didziokas et al. (2024a). In the interest of reducing the number of animals sacrificed, it was deemed that an increased number of animals was not required (in line with principals of 3Rs). However, the low number of animals included in this study represents a limitation of this work.

Note: All animal experiments were approved by the UK Home Office and performed as part of a Project License (number: PP8161503) under the UK Animals (Scientific Procedures) Act 1986. Animal procedures complied with the ARRIVE guidelines and were performed under the supervision of UCL Biological Services.

### Loading setup

Static loading was carried out following the previous study that originally mimicked the dynamic loading setup from Moazen et al. (2022). The animals were placed in a simplified anaesthetic tube with the same contact point preserved as in the in vivo loading. 10 g (0.1N) weight was then placed on the plunger attached to a 1 mm diameter tip freely positioned across the skull. The loading was carried out in a micro-CT scanner (XT H 225ST, Nikon, Herts., UK) and the specimens were scanned before and during the loading. All the CT scans were taken at 90 kV for 2 h with a voxel size of 9.5 µm. Each animal was only used for one loading.

Initially, a load relaxation test was carried out which identified 120 min as the required time for the relaxation (see Didziokas et al. 2024a). However, during the development of this study, the significantly larger deformation of the other loading locations led to a significantly longer relaxation period. At P7 12 hours were required for the specimen to fully relax under the load, at P14 4 h and at P21 the original 2 h were sufficient. While the relaxation times were different it should be noted that past this time no deformation occurred, for example, the frontal P7 loading deformation remained the same after 12 h as it was after 2 h. Thus, all of the animals were scanned at the same state of cessation of deformation under a 0.1 N load.

### Computational models

**Fig. 2:**
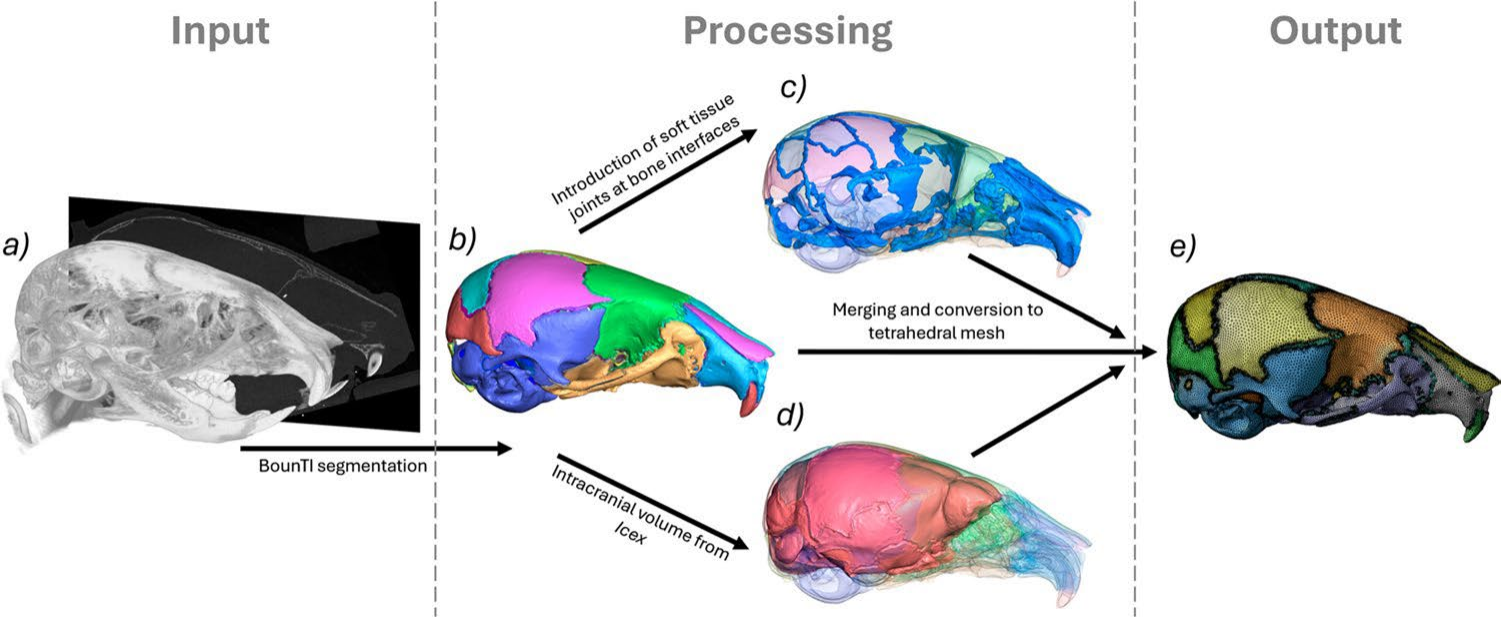
Rapid computational model development methodology. **a** CT data (P7, P14, P21 WT), **b** fully disarticulated segmentation of the craniofacial system using BounTI (Didziokas et al. 2024a, b), **c** generated soft tissue joints from the contacts regions of the separated bone, **d** extracted intracranial volume using Icex (Buzi et al. 2023) and **e** combined and tetrahedrally meshed model

The computational models were developed using a novel rapid development workflow presented in Fig. 2. The unloaded scans of the frontal bone loading WT cases were used for the generation of the computational models. A novel automated segmentation tool BounTI (Didziokas et al. 2024b) was used to obtain the fully disarticulated segmentation of the craniofacial system (Fig. 2b) for the three specimens. With all of the specimens scanned using the same parameters, the same BounTI parameters were used to produce the segmentations (number of segments—51, number of iterations—100, initial threshold—19,000, target threshold—17,000).

To generate the sutures the disarticulated segmentation was expanded 5 voxels where the majority of the bones were in full contact with the adjacent bones. Expansion of 5 voxels produced the best results as it ensured the contact of all the bones, but did not over expand the bones. The contact voxels were assigned to sutures. These suture segments were expanded by 5 voxels and imported to the disarticulated segmentation. A series of smoothing and expansion only on the suture segments were carried out (smoothing of 9 voxels, expansion of 1 voxel, smoothing 9 voxels) until the suture segmentation followed the shape of the skull smoothly and did not extrude over the bone while at the same time capturing the full depth of the suture. (Fig. 2c).

The disarticulated segmentation was then converted to a tessellated mesh. Smoothing of 3 voxels was applied in Avizo (Thermo Fisher Scientific, MA, USA) and the mesh was reduced to 500,000 surface triangles. Using Icex (Buzi et al. 2023), an R package for the extraction of endocast, the intracranial volume was extracted based on the surface mesh of the skull. (Fig. 2d) The intracranial volume mesh was then converted to segmentation and combined with the disarticulated segmentation of the skull. Additionally, the intracranial volume surface was non-rigidly aligned to the outside surface of the skull. This was used to generate the covers at the larger gaps in the skull such as the foramen magnum, which were later assigned the same material properties as the sutures.

Lastly, the combined segmentation was again smoothed with the extent of 3 voxels in Avizo and the generated surface mesh was reduced to 500,000 surface mesh and remeshed with a high regularity objective (vertex valence option of 3 passes and triangle quality option of 30 passes). The process was identical to the generation of bone surfaces for strain estimation with the only differences of inclusion of the intracranial volume and more bones (only the frontal, parietal, interparietal and lambdoid bones were included for the strain estimation). The generated surfaces were imported to ANSYS SpaceClaim (ANSYS Inc., Canonsburg, PA, USA), where they were converted to parametric bodies and a tetrahedral mesh was generated in the mesh module. (Fig. 2e) Again the meshing parameters were identical to those for the strain analysis originally established in Didziokas et al. (2024a). Adaptive size function was used for the mesh with the maximum set to 0.2 mm resulting in the average element size in the bone of 0.1 mm and 0.03 mm for the sutures. This represented at least 2 elements over the thickness of the bone and at least 3 elements over the bone-to-bone direction of the sutures.

The models were then displacement-constrained at the maxillary incisors, i.e. contact with the anaesthetic tube and basioccipital bone where most posterior ventral nodes were fixed in all degrees of freedom. The forces were applied on a 1 mm diameter disc of nodes in locations corresponding to the experiments on the frontal, parietal (anterior and posterior regions) and interparietal bones in the dorsoventral direction. (see Fig. 1c for the loading locations) All material properties investigated were elastic isotropic and a consistent Poisson’s ratio of 0.3 was used. The Young’s modulus was varied for the different scenarios investigated. For the brain value, 150 MPa and 3 Pa were used, for the sutures values of 30 MPa and 30 kPa were used and the values for bone ranged from 7000 MPa to 10 MPa. The reasoning behind the investigation of these specific values is detailed in the following section of the methodology, i.e. 2.4 Analyses. A significant limitation of this work is the use of linear isotropic elastic material properties for all of the constituent parts of the model, however, no other values were available in the literature, especially for the early developmental time points investigated here. This limitation is extensively discussed later on.

### Analyses

*Coronal suture gap (P7 WT and MT):* To understand the impact of the external location varied loading on the coronal suture, the coronal suture gap between the frontal and parietal bones was measured using the micro-CT data. The gap was measured instead of the previously laid out suture thickness measurements in Didziokas et al. (2024a) to allow comparison between MT and WT animals. The measurements were taken every 10 slices over 200 slices (in the mediolateral plane) with only the straight part of the coronal suture included on each side (i.e. left and right coronal suture) the measurement was taken from the dorsal edge of the frontal bone to the dorsal edge of the parietal bone (Fig. 1e). The analysis was carried out for both WT and MT animals of the four investigated loading conditions at P7. The MT measurements were only taken where the gap was present, no 0 mm gaps were included in the data, but were present in the slices with complete fusion. The unloaded and loaded states of the same animal were compared. Note: the voxel size was 9.5 µm, and the measurement ranged from 9 to 19 times the voxel size for the unloaded WT and from 4 to 9 times for the unloaded MT.

*Mechanical strain across the sutures (P7, P14 and P21 WT):* To quantify the level of mechanical strain induced across the calvarial sutures due to varied loading location the methodology developed and validated in Didziokas et al. (2024a) for strain analysis was used in this work. In brief, the unloaded bone surfaces were aligned to the loaded bone surfaces from which the strain in the sutures was estimated. The only departure from the previously developed methodology was the use of a non-rigid deformation algorithm by Li and Harada (2022) known as the neural deformation pyramid instead of the previously proposed element-based rigid alignment. This was changed as the bone experienced significantly higher local deformations during the more parietal loading conditions than the frontal bone loading originally investigated. Additionally, with the development of an automatic segmentation tool BounTI, it was now possible to carry out the same analysis but include more sutures. Thus, while originally only the coronal and sagittal sutures were included, here the strain investigation was expanded to include interparietal as well as the lambdoid sutures. This methodology enabled estimation of the mechanical strain across the individual sutures (in response to external loading on the skull bones) for which the von Mises as well as 1st and 3rd principal strains were recorded.

*Comparison of computational and experimental strain across the sutures (P7, P14 and P21 WT):* To investigate the sensitivity and validity of the computational models their results were compared with the experimental data considering the choice of key input parameters to the models. Here, three different conditions were investigated for the brain and suture material properties. Originally Young’s moduli of 150 MPa and 30 MPa for brain and suture, respectively, were used in line with the computational study carried out by Marghoub et al. (2019). However, the physical phenomena estimated in the previous study were different to the external loading considered here. Thus, the high brain Young’s modulus was deemed inappropriate. Previously 10 MPa value was used by Moazen et al. (2022), however, the literature suggests that the linear elastic material model may be unreliable, especially at large deformations as observed here. (Kaster et al. 2011; Mihai et al. 2015; MacManus et al. 2016; Voyiadjis and Samadi-Dooki 2018) Thus, instead of optimising various coefficients for a hyperelastic model, it was decided to discount the brain contribution entirely using a linear elastic isotropic model with Young’s modulus of 3 Pa. The effect of suture Young’s modulus was investigated and 30 kPa (as opposed to 30 MPa) was found to produce overall directional displacement of all the bones individually closest to the experimental results at P7 with the adjusted brain modulus. Values used for the bone were: 3500 MPa, 5100 MPa and 6700 MPa for P7, P14 and P21, respectively, estimated from Moazen et al. (2015). The three cases described here were then compared to the experimental results at the 4 different loading locations for the 3 different ages in WT animals.

*Bone Young’s modulus optimisation (P7 WT optimisation and P7, P14 and P21 comparison):* To investigate the effect of the bone material properties that were left unaltered in the previous comparison additional analysis were carried out. Here the bone Young’s modulus was adjusted from 10 to 7000 MPa for the P7 case (3500 MPa used in the previous investigation). All of the loading locations were investigated and the magnitude of the percentage difference in average strain across the suture was recorded for each investigated suture individually and all the sutures together. The optimal value for most cases was then investigated following the same comparison from the previous investigation for all of the considered ages.

### Statistical analysis

Statistical analysis was performed in SPSS (IBM SPSS, NY, USA). One-way analysis of variance (ANOVA), with Levene’s test was used to test for equal variances. The significance level was set at *p* < 0.05.

## Results

### *Qualitative loading comparison* (P7, P14 and P21—MT and WT)

**Fig. 3:**
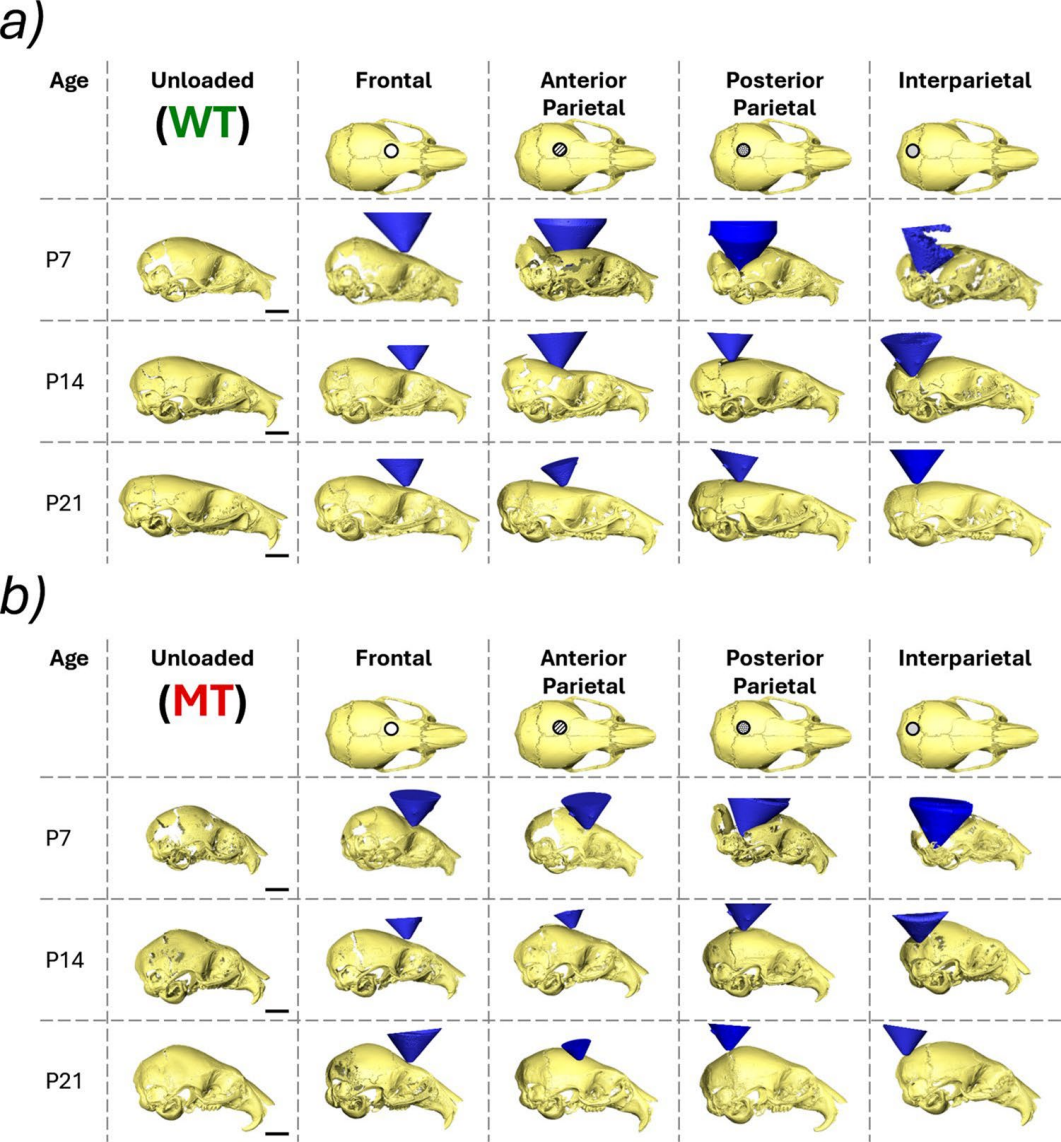
Reconstructions of deformed skull shapes of each investigated individual at 3 different ages (P7, P14 and P21) at 4 different loading locations (frontal bone, anterior part of the parietal bone, posterior part of the parietal bone and interparietal bone) and the loading tip (blue cone). **a** Wild-type animals and **b** mutant type animals

The reconstructed loaded skulls including the loading tip are shown in Fig. 3a for WT and Fig. 3b for MT animals. Firstly, for both the mutant- and wild-type animals the overall deformation of the skull during the frontal bone loading appeared to be the mildest compared to the other 3 investigated locations at P7. Additionally, the level of deformation seemed to increase the more posteriorly the skull was loaded. In general, the overall deformation of the skull across all considered locations decreased with age. However, this was more pronounced in the mutant animals where P7 animals experienced extreme levels of deformation but P14 remained relatively undeformed under the 10 g load. This was not the case in the WT animals where large deformations were still present at P14. For both WT and MT animals, the deformation was significantly lower at P21.

### *Coronal suture gap* (P7—WT and MT)

**Fig. 4:**
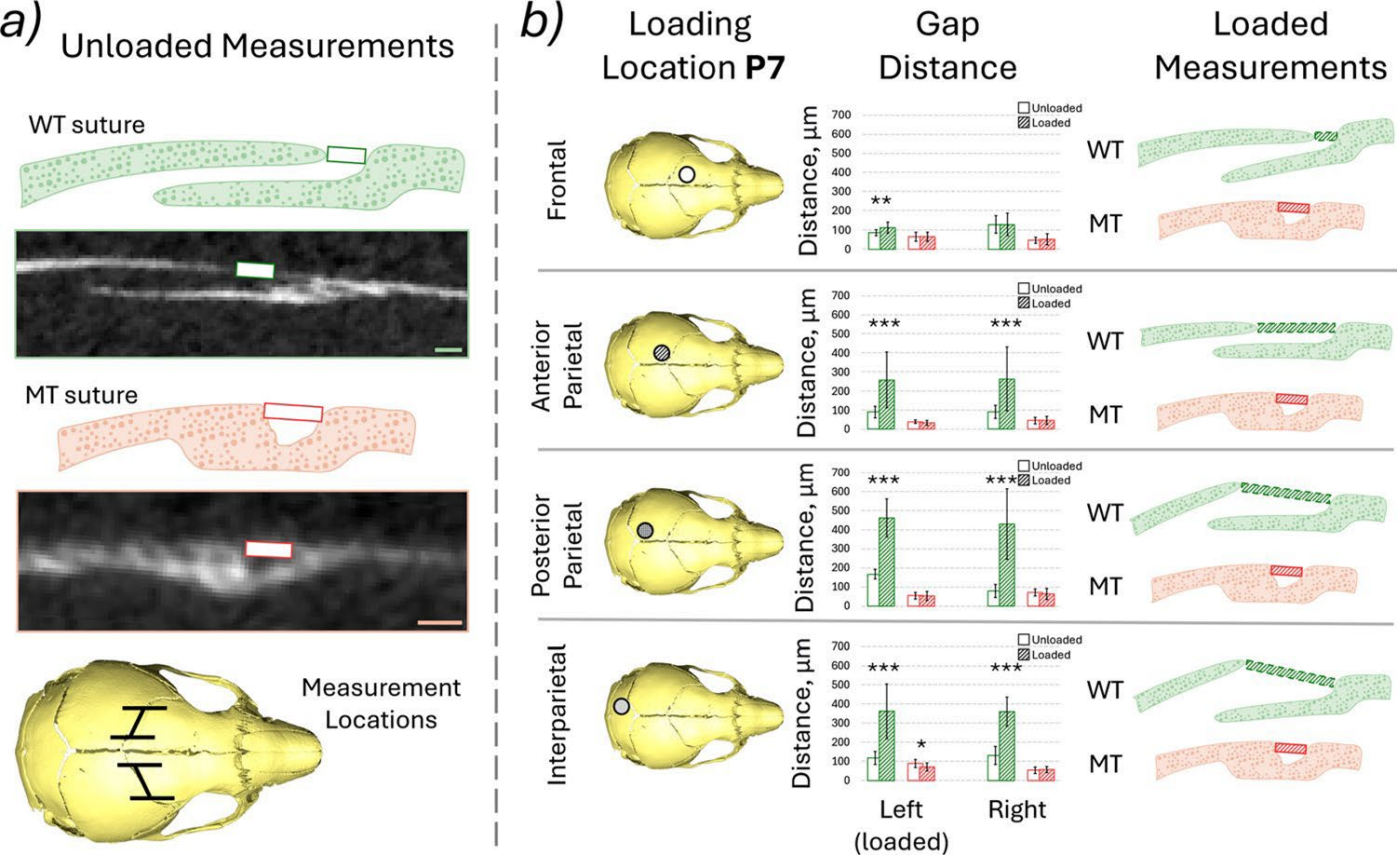
**a** Measured distance and the region measured, 20 slices were investigated in each case every 10 slices of the 200 slices across the straight region of the suture at P7 for both WT and MT animals. Voxel size = 9.5 µm and **b** highlighted loading locations, measured gap distance, green—WT, red—MT, empty box—unloaded and hashed box—loaded, qualitative representation of the deformation at the suture observed. *, ** and *** mark differences between the states at *p* < 0.05, *p* < 0.01 and *p* < 0.001, respectively

The coronal suture gap was measured for all of the loadings at P7 for both WT and MT animals comparing the unloaded and loaded states (Fig. 4b). Both quantitative results as well as representative qualitative sketches of the deformed results are presented in Fig. 4. These data highlighted that:

1. The increase in the coronal suture gap from the unloaded state to the loaded state was statistically significant in all but one investigated case, the frontal bone loading, right coronal suture for WT animals. Conversely, for the MT animals, the only statistically significant change was a decrease in the gap distance for the interparietal bone loaded animal on the loaded (left) side coronal suture with no statistically significant difference for all the other sutures in all the other loadings.
2. The average statistically significant increases for the WT animals for the left and right coronal sutures, respectively, were 0.33 (*p* < 0.01) and 0 times the original length for the frontal bone loading, 1.89 (*p* < 0.001) and 1.93 (*p* < 0.001) times the original length for the anterior parietal bone loading, 1.76 (*p* < 0.001) and 4.37 (*p* < 0.001) times the original length for the posterior parietal bone loading and 2.13 (*p* < 0.001) and 1.77 (*p* < 0.001) times the original length for the interparietal bone loading. In the MT animals the only statistically significant change was a 0.22 (*p* < 0.05) times decrease of the original length for the left coronal suture in the interparietal bone loading case.
3. Qualitative differences were observed between the deformed suture gaps in the WT animals when the 4 different loading locations were compared. The coronal suture gap measured appeared to remain consistent for the frontal bone loading animal however the gap at the posterior edges of the suture widened. During the anterior parietal loading, the suture thickness remained constant, but the parietal bone moved posteriorly widening the measured gap length. During both posterior parietal and interparietal loading the parietal bone appeared to move posteriorly widening the gap and the overall thickness of the suture increased with the parietal bone edge moving dorsally.

### *Mechanical strain across the* sutures (P7, P14 and P21—WT)

**Fig. 5:**
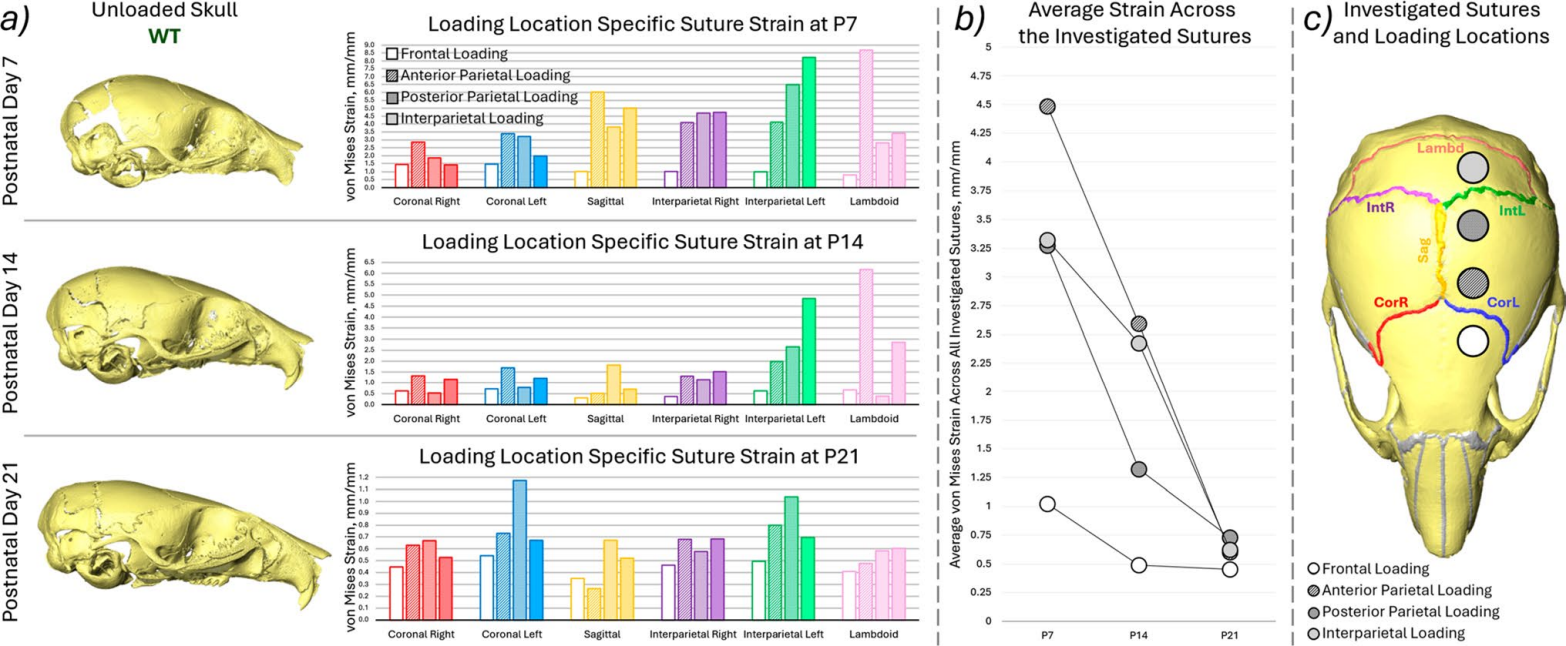
**a** Representative unloaded skull shapes at the three investigated ages (P7, P14 and P21) for WT animals, average von Mises suture strain results for each investigated suture for the 4 different loading conditions (empty box—frontal bone, hashed box—anterior part of the parietal bone, dotted box—posterior part of the parietal bone and solid filled box—interparietal bone), **b** average von Mises strain across all of the investigated sutures compared with age for the 4 different loading conditions, **c** highlighted investigated sutures and the approximate loading locations

The von Mises strain across the investigated sutures (coronal left, coronal right, sagittal, interparietal left, interparietal right and lambdoid see Fig. 5c) as well as the average strain across all the investigated sutures are presented in Fig. 5. Complimentary figures with 1st and 3rd principal strain results are included in Supplement 1. Overall this data highlighted that:

1. The strain across the coronal sutures was higher in all of the investigated loading conditions compared to the frontal loading at all ages. Higher strains were observed in the loaded side sutures (left) compared to the unloaded side for both coronal and interparietal sutures. This pattern was present across all ages and in all loading conditions.
2. At P7 the sagittal, interparietal and lambdoid sutures exhibited higher strains than the coronal sutures. At P14 only the interparietal left (loaded side) and the lambdoid suture showed increased strain. At P21 no prominent increase in strain was observed in the other sutures compared to the coronal suture.
3. The average strain across all of the investigated sutures decreased with age in all of the loading conditions see Fig. 5b. However, while at P21 the strains were comparable for all 4 investigated loading locations at P7 and P14 the strains were markedly higher in the anterior parietal, posterior parietal and interparietal bone loadings compared to frontal bone loading. Similar trends were observed for both 1st and 3rd principal strains in Supplement 1.

### *Comparison of computational and experimental results (P7, P14 and P21*—*WT)*

**Fig. 6:**
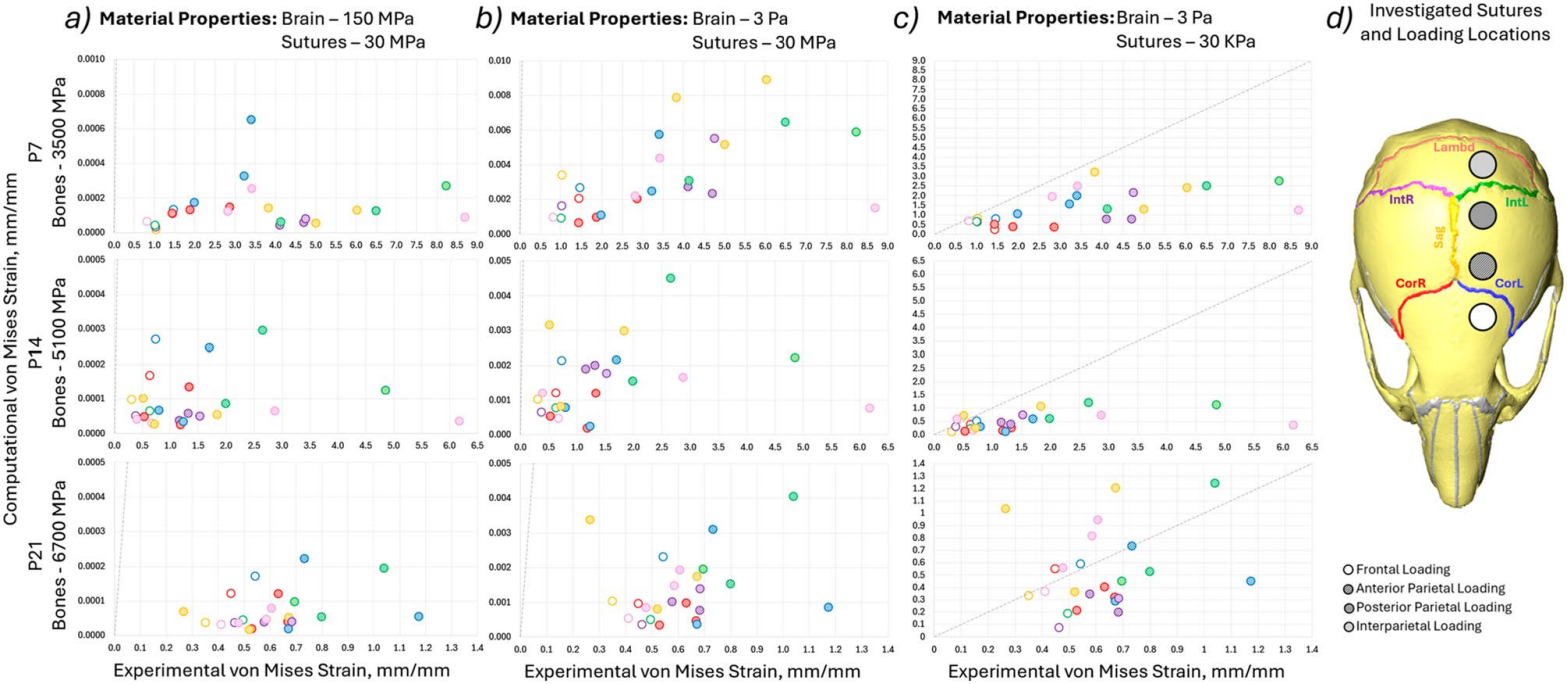
Comparison of the experimental von Mises strain results and experimental von Mises strain results for individual sutures at the 4 different loading locations for 3 different ages (P7—bone Young’s modulus 3500 MPa, P14—bone Young’s modulus 5100 MPa, P21—bone Young’s modulus 6700 MPa). The colours indicate different sutures and the marker types indicate different loading locations (i.e. the same simulation). **a** 150 MPa, 30 MPa, for the brain and suture Young’s modulus, respectively, **b** 3 Pa, 30 MPa, for the brain and suture Young’s modulus, respectively, **c** 3 Pa, 30 kPa, for the brain and suture Young’s modulus, respectively, and **d** highlighted investigated sutures and the approximate loading locations

Computational models of three different ages with varying bone properties were investigated (P7—3500 MPa, P14—5100 MPa and P21—6700 MPa). Three different sets of Young’s moduli were investigated for the brain and suture material properties. (150 MPa, 30 MPa; 3 Pa, 30 MPa; 3 Pa, 30 kPa for the brain and sutures, respectively) The computational von Mises strains were plotted against the experimental von Mises strains across the same sutures in Fig. 6 and Table 1 includes Lin’s Concordance Correlation Coefficient (CCC) (Lin 1989), best-fit line equation and *R*^2^ to the best-fit line. The results highlighted that:

1. With the material properties from literature Fig. 6a and b (Table 1a and b) the computational strains were orders of magnitude underestimated (10,000 times for the 150 MPa brain case and 1000 times for the 3 MPa case). With Young’s modulus for the suture adjusted to 30 kPa, the computational strains were more in line with those obtained experimentally see Fig. 6c.
2. While the computational results were closer to the experimental values for the 30 kPa suture case, they did not significantly correlate with the experimental results with the CCC ranging from − 0.3 to 0.327 for P7, from − 0.0796 to 0.507 for the P14 case and from − 0.5857 to 0.1282 for the P21 model see Table 1. The negative values indicate that as the experimental strain across the individual sutures increases the predicted computational strain for those sutures individually decreases, predicting the opposite pattern. This was observed in the best-fit lines with the negative coefficients of the x terms.

**Table 1:** Statistics for computational to experimental correlation

Age	Results	Frontal	Anterior Parietal	Posterior Parietal	Interparietal
(a) Material Properties: Brain—150 MPa, Sutures—30 MPa					
P7 Bones 3500 MPa	CCC	0	0	0	0
	Y	− 8.2193*x* + 1.0582	− 2054.9*x* + 4.6673	− 638.81*x* + 3.5249	11940*x* + 1.9538
	*R*^2^	0.000003	0.0316	0.001	0.1878
P14 Bones 5100 MPa	CCC	0	0	0	0
	Y	537.25*x* + 1.0594	− 13455*x* + 4.7958	9249.6*x* + 2.8699	61664*x* + 0.8984
	*R*^2^	0.0113	0.325	0.0836	0.6053
P21 Bones 6700 MPa	CCC	0	0	0	0
	Y	397.26*x* + 0.4052	780.91*x* + 0.5101	1418*x* + 0.6554	1274.2*x* + 0.547
	*R*^2^	0.2919	0.09	0.3193	0.3872
(b) Material Properties: Brain—3 Pa, Sutures—30 MPa					
P7 Bones 3500 MPa	CCC	0.0001	0.0001	0.0005	0.0006
	Y	122.2*x* + 3.8386	196.85*x* + 2.7287	449.82*x* + 1.9299	907.52*x* + 0.6857
	*R*^2^	0.0173	0.0412	0.4772	0.8024
P14 Bones 5100 MPa	CCC	0.0002	− 0.0003	0.0011	0.0003
	Y	111.05*x* + 0.4332	− 1770.4*x* + 5.3872	554.47*x* + 0.1148	1399.5*x* + 0.4297
	*R*^2^	0.1466	0.5346	0.9382	0.5967
P21 Bones 6700 MPa	CCC	0.0002	− 0.0004	0.0004	0.0001
	Y	41.472*x* + 0.4106	− 58.495*x* + 0.7008	72.766*x* + 0.6677	48.998*x* + 0.5609
	*R*^2^	0.1977	0.1231	0.1337	0.2127
(c) Material Properties: Brain—3 Pa, Sutures—30 kPa					
P7 Bones 3500 MPa	CCC	− 0.3	0.0894	0.2856	0.327
	Y	− 0.7188*x* + 1.5654	1.3263*x* + 2.5776	0.9474*x* + 1.9306	2.1599*x* + 0.341
	*R*^2^	0.3337	0.1521	0.3288	0.6581
P14 Bones 5100 MPa	CCC	0.2487	− 0.0796	0.507	0.2262
	Y	0.6734*x* + 0.3509	− 2.3652*x* + 2.466	1.7847*x* + 0.0786	3.3186*x* + 0.2977
	*R*^2^	0.3245	0.1432	0.7632	0.7647
P21 Bones 6700 MPa	CCC	0.092	− 0.5857	0.1282	0.0199
	Y	0.0619*x* + 0.4286	− 0.4314*x* + 0.8468	0.0885*x* + 0.72	0.0157*x* + 0.6099
	*R*^2^	0.0342	0.3964	0.0214	0.0028

Lin’s concordance correlation coefficient (CCC), best-fit line (Y), and coefficient of determination (*R*^2^). a) Material Properties: Brain—150 MPa, Sutures—30 MPa, b) Material Properties: Brain—3 Pa, Sutures—30 MPa, c) Material Properties: Brain—3 Pa, Sutures—30 kPa

### *Bone young’s modulus optimisation (P7*—*WT optimisation and P7, P14 and P21*—WT* comparison)*

**Fig. 7:**
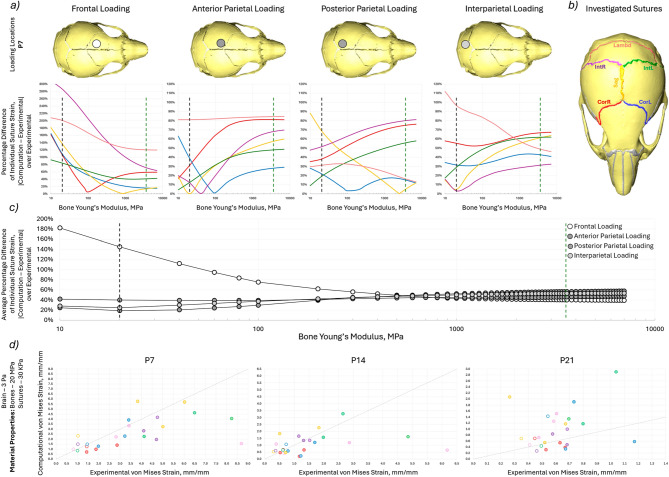
**a** percentage difference of the computation von Mises strain to experimental von Mises strain at P7 for each investigated suture for the 4 different loading conditions with varying bone Young’s modulus. The green dashed line indicates the literature value for the bone Young’s modulus (3500 MPa) and the black dashed line indicates the optimised Young’s modulus (20 MPa), **b** highlighted investigated sutures, **c** average percentage difference of the computation von Mises strain to experimental von Mises strain at P7 across all investigated suture for the 4 different loading conditions with varying bone Young’s modulus. The green dashed line indicates the literature value for the bone Young’s modulus (3500 MPa) and the black dashed line indicates the optimised Young’s modulus (20 MPa) and **d** Comparison of the experimental von Mises strain results and experimental von Mises strain results for individual sutures at the 4 different loading locations for 3 different ages with minimised suture percentage strain difference at Young’s moduli of 3 Pa, 20 MPa and 30 kPa for brain, bone and suture, respectively

The bone properties were investigated by optimising the Young’s modulus at P7 Fig. 7a and c. The computational strain value was compared to the experimental strain values in terms of the percentage difference of the experimental value. The optimal value for most loading cases at P7 (20 MPa) was then used for the three different ages with the same comparison produced as in Sect. 3.4 see Fig. 7d and Table 2. The results showed that:

1. A decrease in the bone Young’s modulus resulted in a decrease in the right coronal and sagittal suture strain percentage difference for the frontal loading, however, the percentage difference increased for the other sutures. For the other loading cases, the percentage difference decreased with lower Young’s modulus for most sutures with the exceptions of the lambdoid suture for the posterior parietal case and for the interparietal case. The exact value for optimal prediction of suture strain varied between loading conditions and individual sutures.
2. The average percentage difference values across all the investigated sutures were compared to find the most applicable Young’s modulus. In the anterior parietal, posterior parietal and interparietal loading cases 20 MPa produced the lowest average percentage difference at 19%, 40% and 25%, respectively, compared to 57%, 46% and 53% for the original Young’s modulus of 3500 MPa. However, the 20 MPa Young’s modulus led to an increase in the percentage difference from 40 to 145% for the frontal loading case.
3. The adjusted 20 MPa Young’s modulus represented a significant increase in the correlation of the computational results to the experimental values at both P7 and P14 and minimal change at P21. (Fig. 7d, Table 2) At P7 the CCC ranged from 0.0784 to 0.6008, at P14 from − 0.2318 to 0.8759 and at P21 from − 0.1076 to 0.1356. Additionally, the lambdoid suture in the anterior parietal loading and the interparietal left suture in the interparietal loading at P7 exhibited particularly high levels of strain experimentally at 8.689 and 8.222, respectively. The CCC and *R*^2^ for these two loading locations in brackets represent the values with these sutures removed from the statistical analysis.

**Table 2:** Statistics for computational to e*x*perimental correlation

Age	Results	Frontal	Anterior Parietal	Posterior Parietal	Interparietal
Material Properties: Brain—3 Pa, Sutures—30 kPa					
P7 Bones 20 MPa	CCC	0.0784	0.0037 (0.6591)	0.5192	0.6008 (0.8226)
	Y	0.2415*x* + 1.1299	0.0043*x* + 2.9238	0.6576*x* + 0.4721	0.4927*x* + 0.7719
	*R*^2^	0.0145	0.00003 (0.6738)	0.3411	0.6901 (0.8546)
P14 Bones 20 MPa	CCC	0.3651	− 0.2318 (− **0.3077)**	0.8759	0.3532 (0.3576)
	Y	0.5551*x* + 0.3554	− 0.1481*x* + 1.5345	1.1778*x* + 0.1199	0.3123*x* + 0.2025
	*R*^2^	0.1887	0.3852 (0.1001)	0.9014	0.6132 (0.4207)
P21 Bones 20 MPa	CCC	0.1132	− 0.1076 (− **0.1468)**	0.1356	0.1084 (0.0737)
	Y	2.7474*x* − 0.5722	− 1.1592*x* + 1.8416	1.0924*x* + 0.3363	2.9064*x* − 0.9483
	*R*^2^	0.2028	0.1068 (0.2117)	0.0952	0.1912 (0.0736)

Lin’s concordance correlation coefficient (CCC), best-fit line (Y), and coefficient of determination (*R*^2^). Material Properties: Brain—3 Pa, Sutures—30 kPa, Bone—20 MPa

## Discussion

In our previous study, (Didziokas et al. 2024a) a series of in/ex vivo experiments were carried out to characterise the level of mechanical strain induced across the coronal suture in mutant- and wild-type mice during the external frontal bone loading. This was carried out to understand the therapeutic effects observed in dorsoventral cyclic loading of the frontal bone in coronal suture patency retention observed by Moazen et al. (2022). The study validated a novel suture strain estimation approach during loading and provided insight into the level of strain during external loading laying the foundation for computational studies. However, several questions remained unanswered such as the effect of loading location on suture strain and the level of strain in the coronal suture of the MT animals during loading.

In this study, further experiments were carried out to investigate the effect of loading location on the suture strain and more sutures were included in the analysis than previously. Additionally, the deformation of the suture gap was measured to estimate the level of deformation and strain at the coronal sutures of the WT and MT animals specifically comparing the response to the external load. The expanded experimental investigation was followed by an investigation of computational models. Only the WT animals were included in both the strain analysis investigations and computational models, the same limitations prevented the investigation of MT animals that were present in the previous study. Namely, the fused suture in the Crouzon mice. Additionally, only linear elastic properties were investigated for the material models in the computational models likely representing the most significant limitation of the computational part of this investigation.

Qualitatively clear differences between the responses of the skulls to the external loading at different locations are present. (Fig. 3) Namely, the previously investigated loading location (frontal bone) exhibited a significantly lower level of relative deformation compared to the other investigated loading locations (posterior parietal, anterior parietal and interparietal bones). The significant difference observed can be explained by the anatomy of the skull. The frontal bones are structurally connected to the skull base and are overall part of a more rigid framework. Conversely, the parietal and interparietal bones at P7 are more loosely connected to the rest of the cranial skeleton and even at P14 still have significant gaps (sutures) present at their boundaries.

These anatomical differences and in turn variable rigidity of the skull may be linked to the developmental role the sutures assume. The posterior region of the skull elongates in WT animals from P14 to P21 see Fig. 3a. Potentially, the structure of this region allows this elongation to occur by providing the least resistant growth direction for the brain, but in turn leads to a significantly less stiff structure which is more susceptible to external loading. The mutant animals appear to confirm the link between the external rigidity of the posterior skull and its elongation during development as the posterior region is significantly more rigid in the MT animals at P14 compared to the WT animals and the characteristic elongation does not occur.

The premature fusion of the sutures may account for an early stiffening of the skull to external loading. As early as P7, the frontal and posterior parietal bone loadings appeared to produce minimal levels of deformation compared to their WT counterparts. Suggesting that the affected sutures may play a role in the mechanical response as early as P7. Another significant difference in the mutant loading response appeared to be the local deformation of the bone. The *Fgfr2*^*C342Y/*+^ mutant mice present with decreased mineralisation of the frontal and other bones (Liu et al. 2013; Moazen et al. 2015; Ajami et al. 2024). As the loading locations coincided with the less bony regions the deformations qualitatively appeared to occur across the bone as opposed to across the sutures as in the WT case. The premature fusion at the sutures that was present even at P7 may have further limited the deformability of the sutures.

With these qualitative differences between WT and MT responses to the external loading at the four different locations present, the response of the coronal suture was investigated directly for both MT and WT animals at P7 where the deformations were the greatest and the level of coronal suture fusion was the lowest. Importantly, the suture gap distance did not increase in any of the investigated cases for the MT animals, while the gap distance increased significantly (*p* < 0.01 and *p* < 0.001) for the WT animals in all but one case. The only statistically significant change in the MT animals was the decrease in the gap size on the loaded side for the interparietal loading. The interparietal bone loaded MT also exhibited the most patent coronal sutures and the only statistically significant difference thus, was chosen for further qualitative investigation.

**Fig. 8:**
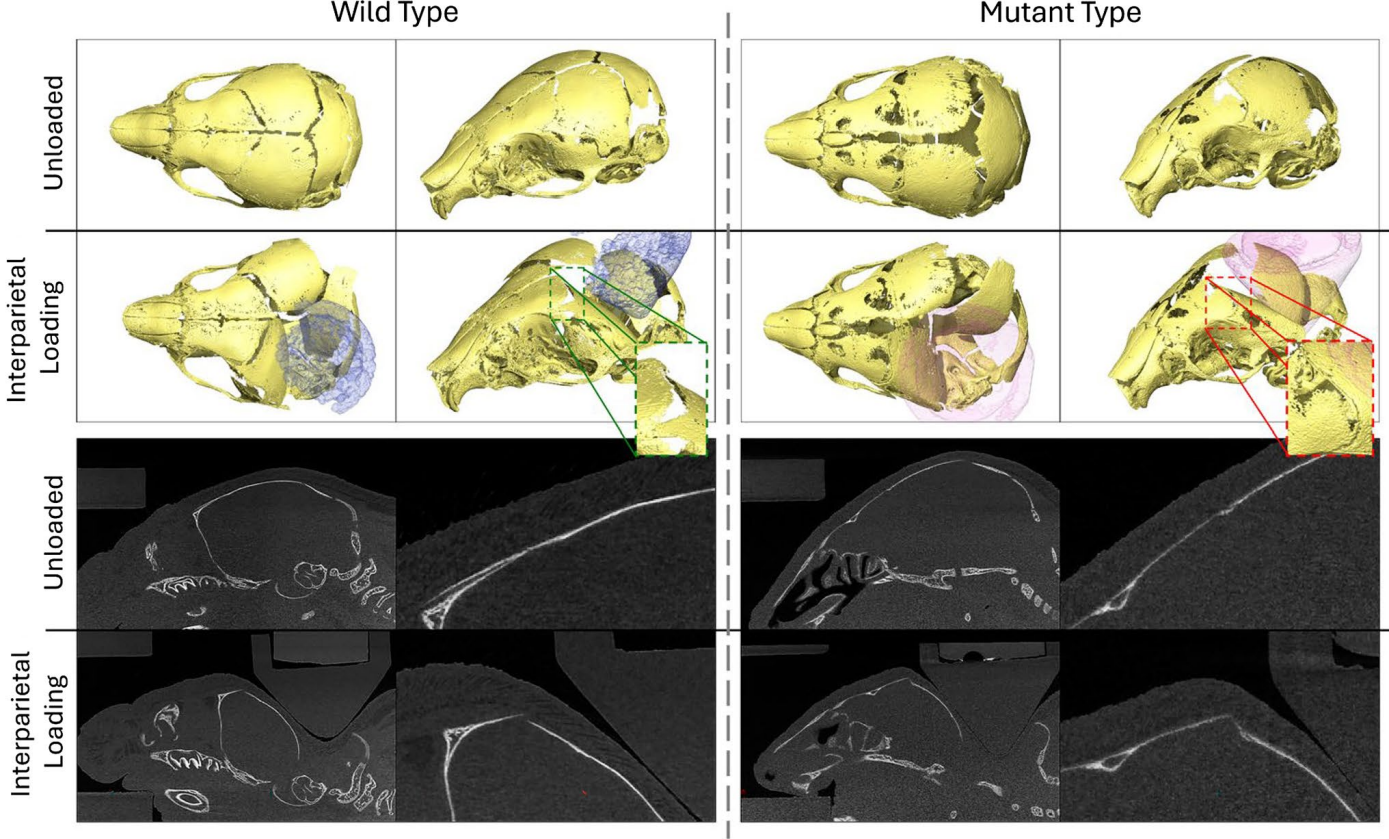
Qualitative comparison of the loaded and unloaded WT and MT skulls during interparietal loading at P7. Highlighting the different responses of the unfused (WT) suture and partially fused (MT) suture to external loading

Figure 8 compares the WT and MT, loaded and unloaded states for the interparietal bone loaded P7 animals. A clear difference in the deformation across the coronal suture was observed in the two cases. Namely, the bones appear to move freely under the load in the WT case, thus changing the sutural gap distance. Conversely, the geometry of the affected suture in the MT case appears to remain unchanged in this case. While the overall deformation of the skull appeared to be comparable in the MT case the deformation did not occur across the sutures and instead appeared to locally deform the frontal bone across the lower ossification region. The lack of deformation across the suture was highlighted quantitatively for the other loading locations and was observed qualitatively during measurement.

These findings highlight the unexpectedly large difference between the response of the coronal sutures to external loading between MT and WT animals. Specifically, no strain in the coronal sutures appeared to be present in the affected animals with the only measurable change being a 22% decrease. Whereas, for the WT animals, the majority of the deformation and therefore strain occurred at the sutures ranging from 33 to 437% increase from the original size. It is then unclear whether the mechanism of suture patency retention proposed by Moazen et al. (2022), where the tensile strain across the sutures maintains the suture patency applies to these animals. As little to no suture strain was observed in the MT animals, especially compared to the extreme changes in coronal suture gap observed in the WT animals. However, the cyclic nature of the in vivo treatments not captured in these investigations may play a significant role in the therapeutic mechanisms. Perhaps, through direct disarticulation of the less affected sutures at early stages of fusion as previously suggested in Didziokas et al. (2024a) or activating mechanotransduction mechanisms differently. However, further investigations of the in vivo treated animals are required to answer these questions.

The early fusion of the sutures in the mutant animals prevented the strain estimation methodology that was originally established in Didziokas et al. (2024a) to be used. Thus, the strain was investigated only in the WT animals at P7, P14 and P21 for the four different loading conditions. In addition to the expanded experimental scope in terms of loading locations, more sutures were included in the analyses than previously (coronal, sagittal, interparietal and lambdoid) see Fig. 5c enabled by the development of BounTI. Overall strain patterns aligned with the patterns observed in the qualitative comparison of the loaded skulls. Namely, the significantly lower strains across the sutures during frontal bone loading compared to the other loading locations. Similarly, a decrease in suture strain was observed across all loading locations with a particularly sharp and even decrease at P21. This suggests that the skull has reached a similar stiffness for all the loading locations investigated as the strains were in range comparing the frontal bone loading to the other loading locations.

The strains across the coronal suture at P7 can be directly compared between the average strain analysis across the full suture and the change in length of the sutural gap. The measured von Mises strains (suture gap change) across the left coronal suture were 1.45 (0.33), 3.39 (1.89), 3.22 (1.76) and 1.97 (2.13), for the loading of frontal, anterior parietal, posterior parietal and interparietal bones, respectively. For the right coronal suture, the values were 1.43 (0), 2.85 (1.93), 1.86 (4.37) and 1.42 (1.77), respectively. The comparison highlights that the values are not the same however, they generally fall within the same range. The suture strain analysis considered the full suture while the gap analysis only investigated the superficial part of the suture in order to compare the results to mutants. This is particularly apparent where strains of 0 were measured for the frontal loading while both qualitatively (in the suture gap analysis) and quantitatively (in the strain analysis) deformation across the suture was observed. Low suture gap change in the frontal loading scenario was observed because the deformation of the bones appeared to hinge around the suture gap see Fig. 4b.

The frontal bone and anterior parietal bone loadings can be considered as “targeting” the left coronal suture as it is the closest suture to the loading locations. Similarly, the posterior parietal bone and interparietal bone loadings can be considered as “targeting” the left interparietal suture. The von Mises strain across the “targeted” suture for the frontal and anterior parietal bone loadings were 1.45 and 3.39, 0.72 and 1.69, and 0.54 and 0.73 for P7, P14 and P21 animals, respectively. The strains across the “targeted” suture for the posterior parietal and interparietal bones were 6.48 and 8.22, 2.65 and 4.85, and 1.04 and 0.69 for P7, P14 and P21 animals, respectively. While at P21 the results across the “targeted” suture were relatively consistent between all the loading locations at P7 and P14 the interparietal left suture, strain was observed to be 2 to 5 times more than the coronal suture under the same loads. This suggests that for the treatment of other sutures by loading different bones the loading parameters established for coronal suture patency are likely inappropriate. The force should be adjusted to account for the age-variable rigidity of the posterior skull.

The average von Mises strains of the six sutures investigated for four different loading locations at three different ages provide a solid foundation for the development of computational models and their validation. Three computational models were developed from the unloaded scans of the frontal bone loading case in WT at the three investigated ages (P7, P14 and P21). Each was loaded on the four different locations investigated experimentally and the computational predictions were compared directly to the experimental values of the von Mises strain. With the higher Young’s modulus values for the suture for both cases (150 MPa, 30 MPa and 3 Pa, 30 MPa, for brain and sutures, respectively) the computational results were orders of magnitude off the experimentally observed values. It should be noted here that the computational models lack skin, muscles and adjoining structures such as the mandible or the spine. However, the inclusion of these structures could not account for the 1000 to 10,000 times lower computational deformation compared to the experimental results and would likely only further marginally decrease the local deformation of the cranial bones. Thus, the discrepancy between the computational and experimental results suggests that the material properties of the sutures in the computational models could not capture the deformation observed physically. Hence, the suture properties were optimised to best match the individual bone directional displacement and the value of 30 kPa was found.

While the computational results with the adjusted suture Young’s modulus were generally in the same order of magnitude as the experimental results they still did not correlate well (highest CCC was 0.507). Tensile tests of the sagittal suture in adult mice were previously carried out by Chien et al. (2008), where a Young’s modulus of 580 kPa was reported for the aforementioned suture. This is significantly closer to the 30 kPa optimised value than the 30 MPa measured using nanoindentation by Moazen et al. (2015). Additionally, the mice investigated by the tensile study were adults and the material properties of neonatal mice are expected to be lower. This highlights that the range of material property studies for the sutures in mice specifically at early ages may be insufficient to fully capture the complexity of the loadings investigated. Lastly, the bone properties were investigated. As large local deformations of the bones were observed qualitatively and were particularly prominent at P7.

The bone Young’s modulus ranging from 3500 to 6700 MPa did not permit any significant local deformation of the bone under the 0.1 N load that was considered in this study. To test the effect of bone’s Young’s modulus on the computational results the value was varied for the P7 models from 10 to 7000 MPa. The investigation highlighted that Young’s modulus of 20 MPa appeared to produce the best strain predictions for all but the frontal bone loading. Again highlighting, that perhaps the frontal part of the skull was significantly stiffer and responded differently when compared to the more posterior part of the skull. The adjusted bone Young’s modulus produced a better correlation with the experimental results in both P7 and P14 animals and did not affect the correlation at P21 see Table 2. While the correlation improved following all the adjustments the predictive power of the models is still questionable.

It should be noted that the investigation of different loading locations was partially inspired by first investigating an optimised frontal bone loading FE model to gauge whether the same material properties would translate to other loading locations. However, the skull appeared to respond differently to external loading with changing loading locations. These differences were hardly captured by the adjusted linear elastic material model of 3 Pa, 30 kPa and 20 MPa for the brain, suture and bone Young’s moduli, respectively. The most significant limitation of the computational part of this work is the linear elastic properties as the brain is known to behave hyperelastically, especially during large deformation. Similarly, the sutures were shown to experience plastic deformation during this loading in the previous study (Didziokas et al. 2024a) and the complex microstructure of the sutures may require a significantly more complex material or computational model to capture. This work highlights the limitations in the understanding of the material properties of the mice craniofacial system, specifically during external calvarial loading early in development.

Currently, little trust can be put in the predictive power of the computational models developed here. Originally, the other loading locations were to be used as validation of the optimised model. However, the experimental evidence showed previously unknown local differences. We suggest, that material properties, specifically focusing on the suture and brain in WT animals and potentially locally variable bone properties in MT animals may be key to developing truly robust FE models of the skull. Further work currently underway has employed similar techniques and developed FE models for pig skulls under external loads, preliminary data from the investigations suggest that linear elastic models may be more applicable in larger animal models where the loads produce significantly lower levels of deformation. Additionally, while new techniques like deep learning-based modelling (Odot et al. 2022) may help capture some of the complexity of the craniofacial systems, it is unclear whether sufficiently large data sets are possible with such time-consuming and expensive experiments. Further material characterisation may remain the best step forward.

A significant limitation in this work was the omission of soft tissues (muscle, skin) from the FE models. As the FE models were shown to produce orders of magnitude lower deformation compared to experimental results when using material properties available in the literature it was chosen to omit the other soft tissues. The likely primary effect of the inclusion of the other soft tissues would be a further reduction in the deformation. Additionally, all the same issues of using elastic linear material properties would be present for the other soft tissues, in the presented experimental data, the soft tissues above the skull compresses (or shifts) from 1 mm thickness to 67 µm thickness (93% reduction) under the tip over the frontal bone loading location. Equally, the mouse skin is not directly attached to the loading locations considered and can easily shift around the skull, modelling such behaviour would introduce further complications. Still, the omission of the soft tissues may introduce further differences between the experimental and FE results as the soft tissues are implicitly present in the experimental work where fully intact mice were used.

For this study FE model development process itself represents a significant investigation into the mechanical behaviour of the mouse skull. The experimental investigations produced in this work can help further the treatment developed by Moazen et al. (2022) and provide insight into craniofacial morphogenesis. Namely, the investigation into the suture gap represents the first direct measurement of the deformation of the coronal suture during external loading in Crouzon mice. Surprisingly, highlighting no measurable tensile deformation of the suture, that was previously assumed to contribute to the retention of suture patency. Additionally, the apparent variable stiffness of the skull to external loading with more deformation observed posteriorly could explain the mechanism of the posterior skull elongation observed from P14 to P21 in mice. With the lower stiffness providing the least resistant path for the brain to expand during growth thus, resulting in the elongation of the skull. Lastly, the investigation into the strain across the other sutures suggests that the same loading force is likely inappropriate at P7 or P14 to target the fusion of interparietal sutures and should be appropriately reduced.

In summary, this study expanded on the previously reported suture strains during external loading, by including more sutures (coronal, sagittal, interparietal and lambdoid) and by investigating different loading conditions (frontal, anterior parietal, posterior parietal and interparietal bones). Computational models were built and compared to the experimental results. In the future, more robust material models specifically for the sutures and brain should be developed and investigated against the experimental results presented in this work to achieve truly predictive computational models.

## Supplementary Information

Below is the link to the electronic supplementary material.

Supplementary file 1 (DOCX 6029 kb)

## Data Availability

Data are provided within the manuscript or supplementary information files.
