# Supplementary material for: Regional variability in craniofacial stiffness: a study in normal and Crouzon mice during postnatal development
Source: Biomech Model Mechanobiol. 2025 May 25;24(4):1207–22. doi: 10.1007/s10237-025-01962-7 (PMC12246019; doi:10.1007/s10237-025-01962-7)
Supplement: Supplementary file 1 — (DOCX 6029 kb) [file 10237_2025_1962_MOESM1_ESM.docx]

**Supplementary materials**

Supplement 1


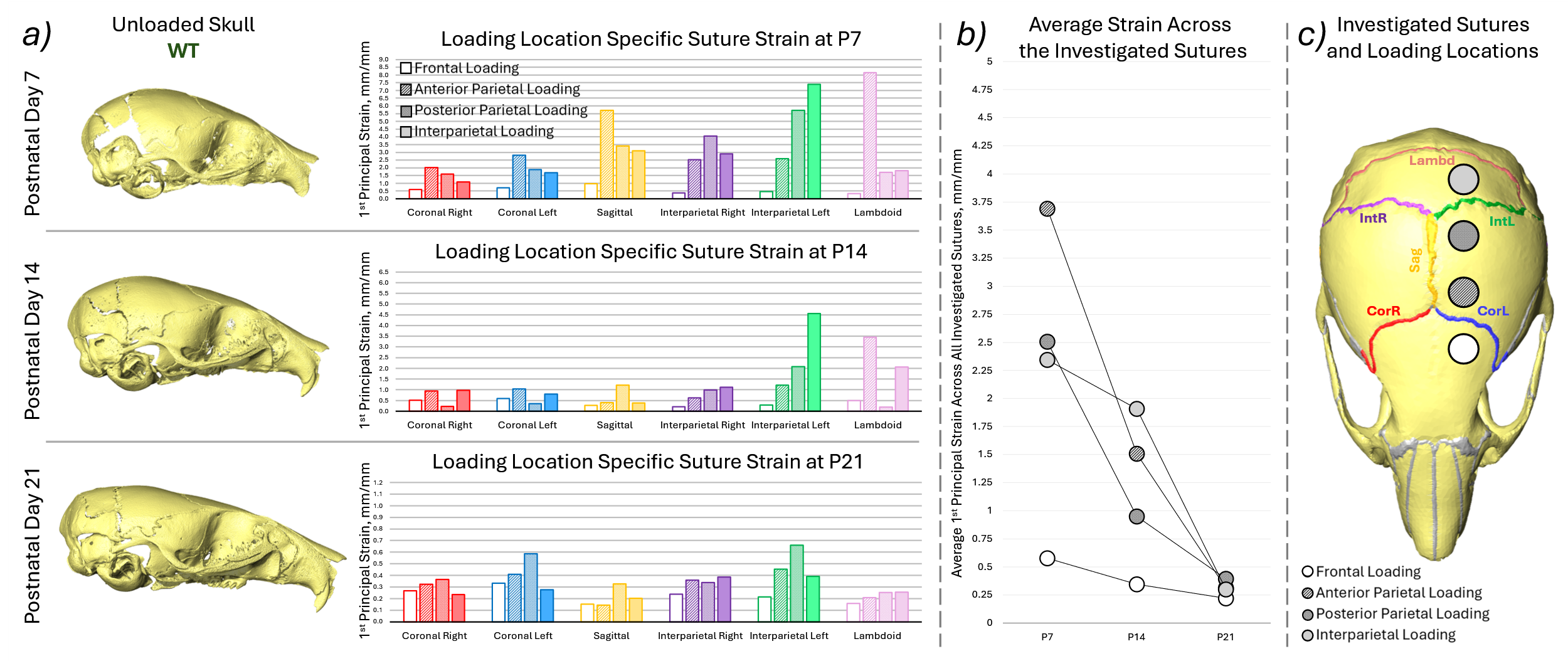


Fig. S1. a) Representative unloaded skull shapes at the three investigated ages (P7, P14 and P21) for WT animals, average 1^st^ principal strain suture strain results for each investigated suture for the 4 different loading conditions (empty box – frontal bone, hashed box – anterior part of the parietal bone, dotted box – posterior part of the parietal bone and solid filled box – interparietal bone), b) average 1^st^ principal strain across all of the investigated sutures compared with age for the 4 different loading conditions, c) highlighted investigated sutures and the approximate loading locations.


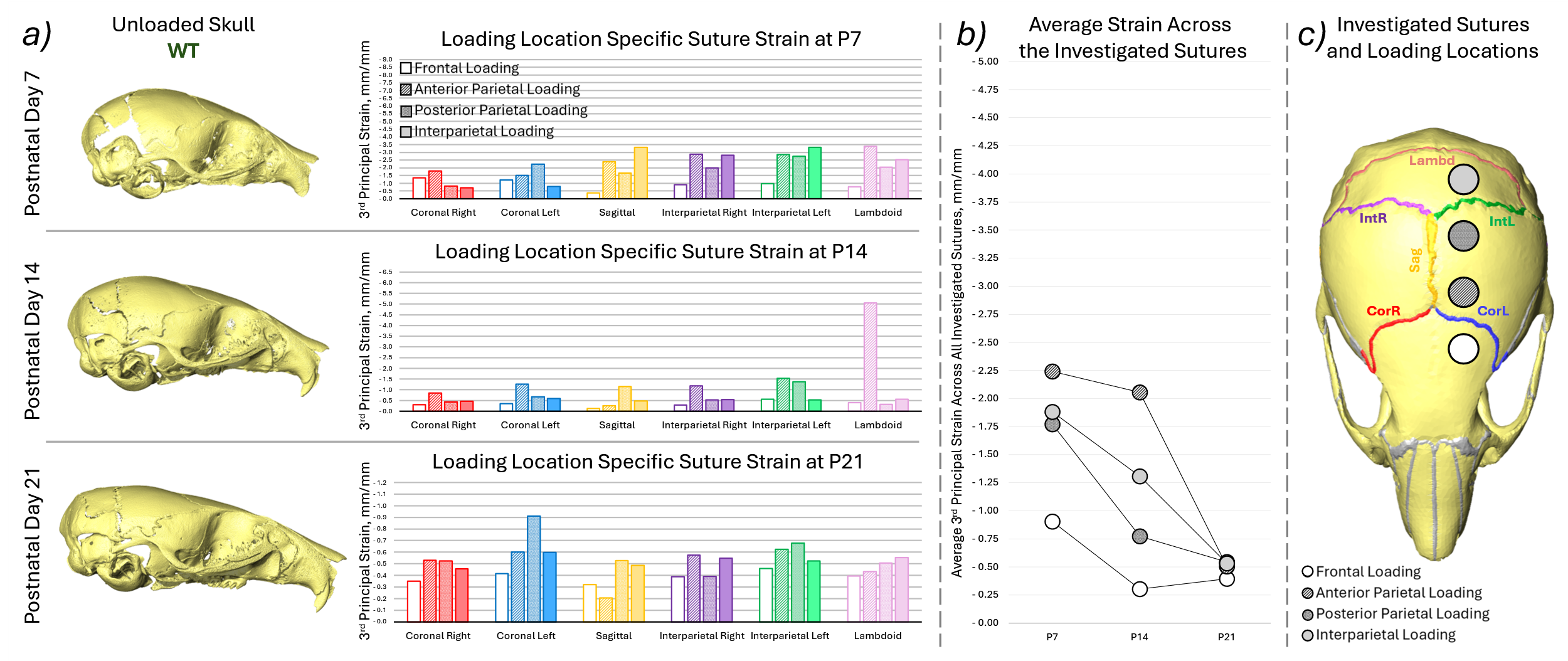


Fig. S2. a) Representative unloaded skull shapes at the three investigated ages (P7, P14 and P21) for WT animals, average 3^rd^ principal strain suture strain results for each investigated suture for the 4 different loading conditions (empty box – frontal bone, hashed box – anterior part of the parietal bone, dotted box – posterior part of the parietal bone and solid filled box – interparietal bone), b) average 3^rd^ principal strain across all of the investigated sutures compared with age for the 4 different loading conditions, c) highlighted investigated sutures and the approximate loading locations.
